# Glutamine Synthetase in Legumes: Recent Advances in Enzyme Structure and Functional Genomics

**DOI:** 10.3390/ijms13077994

**Published:** 2012-06-28

**Authors:** Marco Betti, Margarita García-Calderón, Carmen M. Pérez-Delgado, Alfredo Credali, Guillermo Estivill, Francisco Galván, José M. Vega, Antonio J. Márquez

**Affiliations:** Department of Vegetal Biochemistry and Molecular Biology, Faculty of Chemistry, University of Seville, Apartado 1203, Sevilla 41071, Spain; E-Mails: marbioq@us.es (M.G.-C.); cmperez@us.es (C.M.P.-D.); alfredocreda@us.es (A.C.); gestivill@us.es (G.E.); galvan@us.es (F.G.); jmvega@us.es (J.M.V.); cabeza@us.es (A.J.M.)

**Keywords:** glutamine synthetase, *Lotus japonicus*, functional genomics, nitrogen metabolism

## Abstract

Glutamine synthetase (GS) is the key enzyme involved in the assimilation of ammonia derived either from nitrate reduction, N_2_ fixation, photorespiration or asparagine breakdown. A small gene family is encoding for different cytosolic (GS1) or plastidic (GS2) isoforms in legumes. We summarize here the recent advances carried out concerning the quaternary structure of GS, as well as the functional relationship existing between GS2 and processes such as nodulation, photorespiration and water stress, in this latter case by means of proline production. Functional genomic analysis using GS2-minus mutant reveals the key role of GS2 in the metabolic control of the plants and, more particularly, in carbon metabolism.

## 1. Introduction

### 1.1. Nitrogen Assimilation and Remobilization in Legumes

Nitrogen (N) is one of the most important nutrients for plants and, in natural soils, its availability is often a major limiting factor for plant growth. Of all the mineral nutrients, N is required in the largest quantities for the construction and maintenance of plant cells. Protein contains on average about 12% N by weight, and the whole dry matter of herbaceous plants typically contains from 1.5 to 4.5% N. This explains why 85–90 million tons of nitrogenous fertilizers are added to the soil worldwide annually [1]. Nitrogen is one of the most expensive nutrients to supply and commercial fertilizers represent the major cost in plant production. Furthermore, there is a serious concern regarding nitrogen loss in the field, giving rise to soil and water pollution. Incomplete capture and poor conversion of nitrogen fertilizer also causes global warming through emission of nitrogenous oxide. Lowering fertilizer input and breeding plants with better nitrogen use efficiency is one of the main goals of research on plant nutrition [2,3]. Thus, nitrogen availability is a particular challenge for plant survival, and different metabolic regulations and interactions have evolved to guarantee the strict economy of this essential nutrient during the plant life cycle.

**Figure 1:**
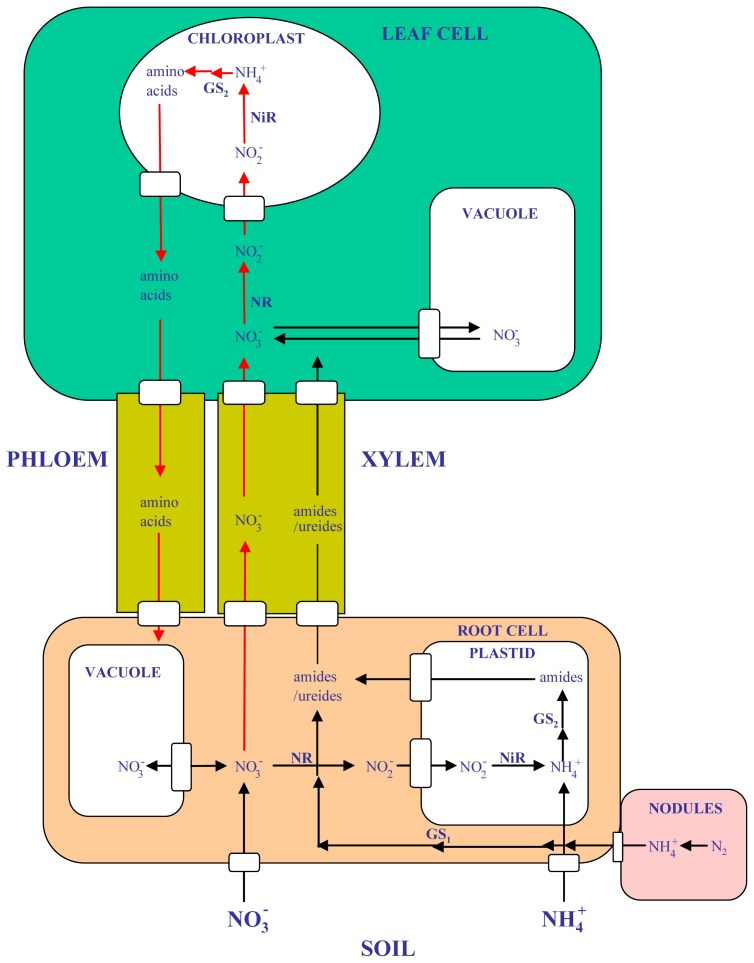
Nitrogen utilization in legume plants. Simple arrows represent single step reaction, while consecutive arrows represent multiple-step reactions. GS_1_, cytosolic glutamine synthetase; GS_2_, plastidic glutamine synthetase; NR, nitrate reductase; NiR, nitrite reductase.

The use of nitrogen by plants involves several steps, including uptake, assimilation, translocation and different forms of recycling and remobilization processes, all of them of crucial importance in terms of nitrogen utilization efficiency [2,3] (Figure 1). Primary nitrogen assimilation by plants involves the use of different forms of inorganic nitrogen (NO_3_^−^ or NH_4_^+^), depending on nitrogen availability, plant species and adaptations. Alternatively, the symbiosis with bacteria enables also to several plant species, most notably legumes, to use atmospheric N_2,_ which is reduced to NH_4_^+^ in the nodules by the action of bacterial nitrogenase. In addition, efficient secondary ammonium assimilation must also exist in plants in order to reassimilate the ammonium ions that can be produced endogenously in the plants from processes such as photorespiration, phenylpropanoid biosynthesis, or amino acid catabolism. Photorespiration is probably the most important process in which high amounts of ammonium are released during the regeneration of 3-phosphoglycerate in the photorespiratory pathway [4]. In fact, the conversion of glycine to serine in the C2 cycle is probably the most important metabolic process that liberates ammonium in photosynthetic cells, at a rate that can exceed by 10-fold the rate of primary assimilation. Glutamine is the first organonitrogen compound that is synthesized in the plants as a result of both primary and secondary ammonium assimilation. Depending on plant species, either glutamine or asparagine [5,6], is the preferred nitrogen compounds utilised to translocate the reduced nitrogen within the plant. Different legume species can use also ureides for N translocation. do Amarante *et al.* [7] have summarized very well our understanding as to when legumes use amides rather than ureides as nitrogen transport compounds in the xylem.

Among the different types of plant species, the Leguminoseae are second only to the Gramineae in importance to humans as a source of food, feed or livestock and raw materials for industry [8,9]. Legumes are the lynch pin of sustainable agriculture because they are able to fix nitrogen in a symbiotic association with *Rhizobium*, which provides these plants and subsequent crops with a free and renewable source of nitrogen (atmospheric N_2_). It is estimated that between 40 and 60 million tons of N are fixed annually by cultivated legumes, which saves about US$ 10 billion in fertilizer [9]. Legumes account for approximately a third of the world’s primary crop production, human dietary protein and processed vegetable oil [8,9]. Legumes are also able to establish beneficial symbioses with soil fungi that enable them to mine phosphorous and other essential nutrients from the soil more effectively. In spite of the importance of legumes in agriculture, increases in yield through breeding over the past few decades have lagged behind those of cereals. Numerous abiotic and biotic impediments continue to limit yield potential in legumes, including: drought, soil salinity, acidity and nutrient limitation; and various diseases and pests. Developing plants that are tolerant to these stresses remains an important aim of breeding programs [9]. While classical plant breeding can and will lead to further improvements in legume phenotypes in the future, the genomics revolution offers alternative and complementary approaches that can aid and accelerate plant breeding. Genomics and functional genomics, together with the more classical scientific disciplines of genetics, biochemistry, physiology, and molecular and cell biology, have already accelerated discoveries in legume molecular and systems biology. Unfortunately, agricultural legumes are relatively poor model systems for genetics and genomics research. Studies on most of the major leguminous crops are hampered by large genome sizes and other disadvantages (polyploidy, transformation or regeneration recalcitrancies, few or large seeds and seedlings, genome duplications, long generation times, *etc*.). As a result, two other species, *Lotus japonicus* and *Medicago truncatula*, have been adopted internationally as models for modern legume research [10–12].

Over the past 20 years, our research group has been working on nitrogen assimilation in the model legume *Lotus japonicus*. A previous review summarized the work carried out on the nitrate assimilatory process in this plant emphasizing the key importance of the root system in this regard [13,14]. In the present work we wish to present a global view of the most recent advances obtained by our group related to glutamine synthetase in this model plant and other legumes.

### 1.2. Glutamine Synthetase and Related Enzymes

Glutamine synthetase (GS, EC 6.3.1.2) is the key enzyme in charge of glutamine biosynthesis in nature. This enzyme catalyzes the incorporation into one molecule of glutamate of the ammonium derived either from primary (nitrate reduction, N_2_ fixation) or secondary forms of nitrogen assimilation, at the expenses of ATP, according to the following reaction: l-glutamate + NH_4_^+^ + ATP → l-glutamine + ADP + P_i_ + H^+^. Two types of GS isoenzymes do exist in plants: cytosolic (called GS1) or plastidic (called GS2). Different GS isoforms, either cytosolic or plastidic, have been reported, which have specific, apparently non-redundant, physiological roles in ammonium assimilation [15–20]. GS1 is localized in the vascular tissue and plays an important role in the assimilation of external ammonium, the ammonia derived from N_2_ fixation and other sources of nitrogen, and in the remobilization of nitrogen during senescence [21,22]. Differential expression of a small multigene family (from 2 to 4 *GLN1* functional genes) is responsible for the behavior of the different cytosolic GS isoforms present in plants [20]. For example, three different cytosolic functional genes plus one pseudogene were found in *Phaseolus vulgaris*, where it was shown that one of this cytosolic genes (GSα) was majoritarily expressed in early developmental stages of the leaves, while a second one (GSβ) is expressed in leaves, roots and nodules, and a third gene (GSγ) is predominantly expressed in nodules coincidently with the onset of the nitrogen fixation process [15]. Conversely, plastidic GS2 is predominantly expressed in green tissues, and it has been demonstrated that this particular isoform has an essential role in the reassimilation of ammonium released by photorespiration [23,24], although its presence in non-photosynthetic tissues of temperate legumes have been also reported [25], as it is the case of *L. japonicus* [24] and *M. truncatula* [26]. Curiously, Taira *et al.* [27] also detected GS2 in *Arabidopsis* mitochondria. However, a single gene encoding for GS2 (*GLN2*) is mostly present in plants, as it happens in the case of the model legume *Lotus japonicus* [28,29], although a second gene encoding for GS2 was recently shown to be exclusively expressed in developing seeds from *Medicago truncatula* [30].

**Figure 2:**
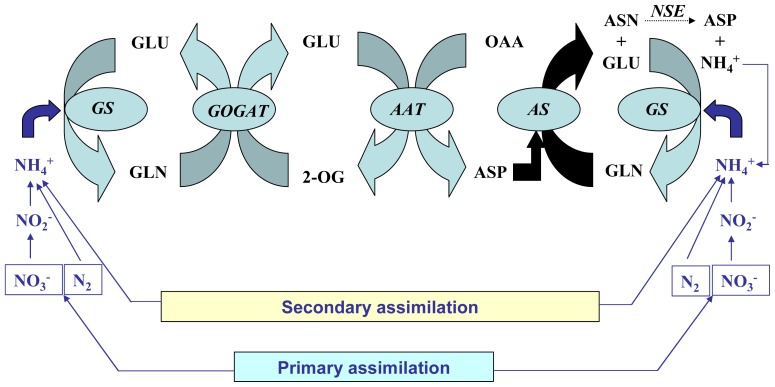
Enzymes involved in glutamine metabolism. GS, glutamine synthetase; GOGAT, glutamate synthase; AAT, aspartate aminotransferase; AS, asparagine synthetase; NSE, asparaginase.

Other enzymes are in charge of the utilization of the glutamine synthesised by glutamine synthetase in order to achieve the synthesis of the rest of organonitrogen compounds required by plants. Glutamate synthase (GOGAT) is of crucial importance because it acts in tandem with GS for the synthesis of glutamate by means of the GS-GOGAT cycle. Two different types of GOGAT enzymes exist in plants called respectively Fd-GOGAT (EC 1.4.7.1) or NADH-GOGAT (EC 1.4.1.14), depending on the use of either ferredoxin or NADH as electron donors [31,32]. Of crucial importance too is asparagine synthetase (AS, EC 6.3.5.4), which acts in conjunction with GS, GOGAT and aspartate aminotransferase (AAT, EC 2.6.1.1) for the synthesis of asparagine that can be used for N translocation in plants (Figure 2). In addition, we should also mention glutamate dehydrogenase (GDH, EC 1.4.1.2) as a complementary enzyme in charge of a reversible amination/deamination reaction which could lead to either the synthesis or the catabolism of glutamate. The role of GDH in glutamate metabolism in plants has been the subject of continued controversy. Except for particular stress conditions most reports indicate that GDH is mostly associated with the catabolic deamination of glutamate rather than a role in glutamate biosynthesis [32].

## 2. Advances in Glutamine Synthetase Research

### 2.1. Enzyme Structure

GS is a ubiquitous enzyme found in all organisms through three different types of proteins: dodecameric GS-I (mostly found in prokaryotes); octameric or dodecameric GS-II (mostly located in eukaryotes), and hexameric GS-III (also found in prokaryotes). GS-I has a M_r_ of around 600,000 and is by far the best characterized of all GS types. The structure of GS-I from several organisms has been determined at atomic resolution [33–36] and it has been found to be a dodecamer built up by two back-to-back hexameric rings. The active site of GS-I, whose residues are conserved in all types of GS, is located between adjacent, intra-ring monomers so that the oligomer possesses 12 active sites containing each one two metal ions (Mg^2+^ or Mn^2+^) that are crucial to the enzymatic activity. Each monomer, with an average length of ~470 residues, is divided in two domains, each contributing to the active site of adjacent monomers. The smaller N-terminal domain contains mostly a sheet made by six antiparallel β-strands of which two form part of the active site. The larger *C*-terminal domain, which is mainly α-helical, contains six β-strands which hold most of the residues building the active site. The dodecamer is maintained mainly by hydrophobic interactions between the two hexameric rings.

**Figure 3:**
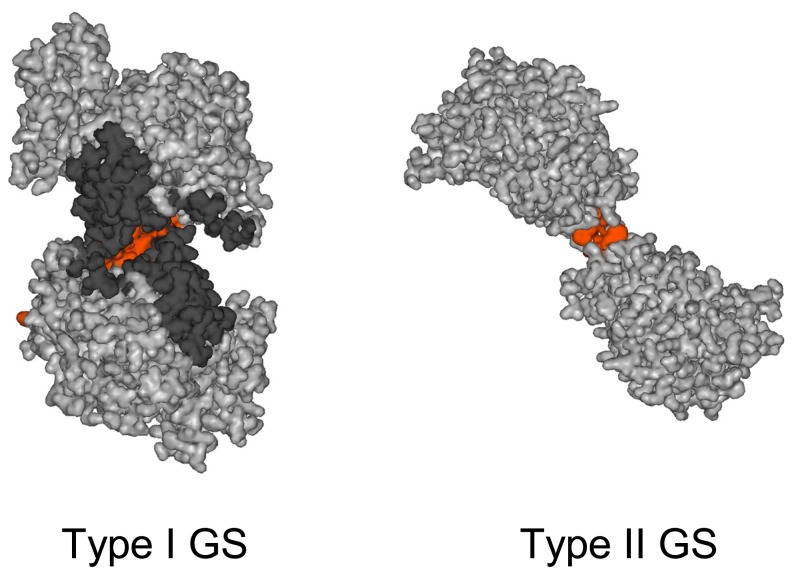
Differences in the amount of inter-ring subunit interactions of type I and type II GS. The figure shows in orange the main contact region among subunits existing in *Salmonella typhimurium* GS (type I GS) compared to GS1a from maize (type II GS) as drawn in a lateral view from their corresponding three dimensional structures.

The GS-II type, a smaller protein than GS-I (~370 residues average length), has been less studied than its prokaryotic counterpart, both in functional and structural terms. Nevertheless, the oligomeric state of GS-II has been the subject of study for more than two decades. Several models have been generated, based on electron microscopy and biochemical studies which point to GS-II as an octamer made by two superimposed rings formed by four identically placed subunits [37–41]. Three-dimensional reconstruction of recombinant homopolymeric α GS-II from *Phaseolus vulgaris* using electron microscopy and image processing, combined with other biochemical and biophysical data, revealed that this protein was an octamer built by two tetramers which are placed back-to-back and rotated 90° with respect each other [42]. The basic symmetry found for the three-dimensional structure (c2) and some biochemical data strongly suggested that the tetramers are formed by the interaction of two preformed dimers so that there are only two active sites per tetramer. The possible existence in this protein of four active sites and four ATP-binding regulatory sites was suggested [42]. Crystallization and X-ray structural determination of GS-II enzyme was achieved by first time for one particular isoform from maize (GS1a), which is remarkably stable [43]. The structure revealed a unique decameric structure and is formed by two back-to-back pentameric rings, with a total of 10 active sites, each formed between every two neighboring subunits within each ring, in a similar form to that described in bacteria, but containing three Mn^2+^ atoms in the middle of the catalytic cleft. A key residue responsible for the heat stability in this protein was found to be Ile-161. More recently, decameric eukaryotic GS II type enzymes from other biological sources have been also reported from humans or animals [44], yeast [45] or *Medicago truncatula* [46]. Remarkable differences between GS-II and GS-I type enzymes are notable in the mode of inter-ring subunit contact. The interface surface between the two pentameric rings of type II GS is much smaller than that of the hexameric rings of bacterial type I GS (Figure 3). This is mainly due to the fact that the primary structure of type II GS has several internal deletions and a *C*-terminal truncation in comparison with the bacterial type I GS. Subunit interaction becomes very limited: only 4 hydrophobic interactions and 2 hydrogen bonds in type II GS1a from maize versus 37 hydrophobic interactions and 36 hydrogen bonds contributed by 43 residues in the case of type I GS [43].

It was determined in our laboratory that one particular cysteine residue (Cys159) of α-GS from *Phaseolus vulgaris* was essential for the enzyme structure and function [47]. Replacement of Cys159 by alanine or serine was sufficient to alter the subunit composition and quaternary structure of the GS enzyme molecule, resulting in a complete loss of enzymatic activity. In addition, treatment of this enzyme with sulfhydryl specific reagents such as pHMB (p-hydroxymercuribenzoate) or DTNB (5,5′-dithiobis-2-nitrobenzoate) confirmed that a strong inhibition of enzyme activity is produced, which is associated with a similar alteration of quaternary structure. Cys159 from *P. vulgaris* α-GS lies within the region equivalent to positions 146–186, that is critical for protein stability in the maize GS1a enzyme with a particular relevance of Ile161 [43]. However, amino acid replacement of Cys159 by alanine or serine in GS1a was not sufficient for the quaternary structure alteration of this protein (Estivill and Hase, personal communication). Other differences among both proteins were observed. For example, the effects of site-directed mutagenesis of Pro146 and Tyr150 on the quaternary structure and enzyme activity of α-GS from *P. vulgaris*, were more intense than those of the corresponding homologue residues in GS1a from maize (Pro146 and Phe50) [48]. These residues in GS1a are mainly involved in the inter-ring subunit interactions [43]. All this suggests that particular differences must exist among the protein structures of different isoforms of higher plant GS, that may be responsible for the observed differences in the quaternary structures (octameric or decameric). While octameric structures may correspond to highly unstable proteins such as the recombinant homopolymeric α-GS from *P. vulgaris*, other decameric structures may correspond to highly stable forms of GS such as GS1a from maize.

Other site-direct mutagenesis studies on the α-GS from *P. vulgaris* were also carried out in our laboratory [49–51], combined with isothermal titration microcalorimetric and fluorescence studies [48,52]. A clear role for Asp56 and Glu297 in the reactivity towards the NH_4_^+^ in the catalytic mechanism of this protein was observed. It was also determined the essentiality of His249 and crucial importance of Arg316 in the interaction with ATP. Thus, the catalytic behavior of highly conserved amino acid residues from the active site of α-GS (type II GS) was found to be similar to that previously reported for type I bacterial GS [33–36]. Interestingly, different effects were produced either on biosynthetic or the transferase catalytic reactions of GS by many of the mutations examined. Therefore it can be concluded that there is a different role of active-site residues on the two catalytic activities commonly assayed by GS researchers. In addition, it was also demonstrated that site-directed mutagenesis of Asp56 residue, which is located far away from the ATP active site compartment, may also affect the ATP binding process. Moreover, different types of results indicate that the affinity for glutamate of the GS protein is highly susceptible to be altered. For example, mutations such as R316Q or D56E may result in important changes in the K_m_ for glutamate in α-GS from *P. vulgaris*. It was also demonstrated the crucial requirement of the metal cofactors in the maintenance of the quaternary protein structure and K_m_ for glutamate of plastidic GS from *L. japonicus* [53]. Other studies carried out in our laboratory have indicated that the K_m_ for glutamate of recombinant higher plant GS enzymes produced in *E. coli* may be substantially different from those of the same enzyme observed in higher plant crude extracts. This is the case of K_m_ for glutamate of recombinant plastidic GS2 from *L. japonicus*, which was found to be 6-fold higher than that from *L. japonicus* crude extracts [28]. All these results suggest the existence of different quaternary structure and/or conformational changes in higher plant GS, which may result either in a tense (low activity due to high K_m_ for glutamate) or relaxed (high activity due to low K_m_ for glutamate) conformation of GS. It is quite likely that different types of post-translational modifications may be responsible for these changes. In fact, different mechanisms of post-translational modifications of plant GS have been reported, involving phosphorylation-dephosphorylation and 14-3-3 protein binding [54–58], thiol regulation [28] and, more recently, sumoylation [59] or tyrosine nitration [60]. The existence of GS post-transcriptional and/or post-translational regulatory events is also suggested by other types of work using transgenic plants [61–64]. Our recent work in collaboration with the group of Dr. Georgina Hernández (ICG, Cuernavaca, México) [48] shows also that very high levels of GS protein expression may not correspond with high levels of enzyme activity in transgenic plants from *L. japonicus*. However, there is not yet a clear idea on how the different post-transcriptional or post-translational regulatory mechanisms may affect the enzyme activity of GS in plants.

### 2.2. Use of Mutants for the Study of Plastidic GS Functionality

The role of the plastidic GS isoform in plants has been historically defined as the reassimilation of the ammonium produced by the photorespiratory cycle [23,24,65,66]. In fact, mutants lacking plastidic GS are conditionally lethal, in the sense that they grow normally under a CO_2_-enriched atmosphere (>0.2%) where photorespiration is suppressed but show serious stress symptoms when transferred to normal air, an atmosphere that permits photorespiration (eventually leading to death if the plants are grown in these conditions for a long time). Photorespiratory mutants were first discovered by Sommerville and Ogren [67]. Different types of photorespiratory mutants were later on isolated that can be impaired in different steps of the C2 photorespiratory cycle. Interestingly, while mutants of some enzymes of the cycle like Fd-GOGAT were isolated in different plant species like *Arabidopsis*, barley, pea and tobacco [65,68–70], mutants of plastid GS were only obtained in barley [23] and *Lotus japonicus* [24], making the latter ones the only GS2 mutants in a legume described so far.

Two different *L. japonicus* plastid GS mutants, initially called *Ljpr1* and *Ljpr2*, were isolated in our laboratory by Orea *et al.* [24] after screening approximately 30,000 plants treated with the mutagen ethyl-methanesulphonate (EMS). Both mutants were allelic and showed a mendelian inheritance of a single recessive trait. The mutants accumulated high levels of ammonium when grown under photorespiratory-permitting conditions and had normal levels of the cytosolic GS isoform. However, chlorosis and necrosis of the edges of the leaves appeared after several days of transfer from high CO_2_ to normal air atmosphere (air-sensitivity phenotype). Mutant plants could be rescued if transferred back to a CO_2_-enriched atmosphere, but long-term exposure to normal air eventually caused the falling of the leaves, beginning from the younger ones. The main difference between the two mutants was that in *Ljpr1* the GS2 polypeptide was present, though in a lower amount with respect to the WT plants, while in *Ljpr2* no GS2 polypeptide was detected. Since both mutants had normal levels of GS1 protein and activity, it was clear that this isoform is not able to compensate for the lack of the plastidic one. Moreover, the similar levels of total GS activity in the leaves of *Ljpr1*and *Ljpr2* (about one third than the WT), together with the similar air-sensitivity of the two mutants, strongly suggested that the GS2 polypeptide detected in the *Ljpr1* mutant lacked enzyme activity. In order to test this hypothesis, and to gain further insight into the functionality of plastidic GS in *L. japonicus*, a molecular analysis of the two mutants was later on carried out [28]. The transcription of the plastidic GS gene was normal in both mutants, indicating that the mutants were affected at the post-transcriptional level. Further sequencing of WT and mutant *GLN2* cDNAs revealed that each of *Ljpr1* and *Ljpr2* carried a different point mutation in one exon of the GLN2 gene encoding for plastidic GS (leading to G85R and L278H amino acid replacements respectively). Since both mutant plants were affected at the level of the *LjGLN2* gene sequence, the mutants were called *Ljgln2-1* and *Ljgln2-2* according to the standard nomenclature [28]. Using different types of approaches, it was demonstrated that the plastidic GS polypeptide from both mutants lacked any detectable enzyme activity. Analysis of the purified mutant enzymes recombinantly produced in *E. coli* indicated that the GLN2-1 mutant polypeptide was assembled into a functional, though unstable GS oligomer. In contrast, the GLN2-2 polypeptide was not able to acquire a proper quaternary structure and was rapidly degraded, in agreement with the different GS2 polypeptide levels observed in extract from leaves of the mutants.

Homozygous *Ljgln2-1 or Ljgln2-2* mutant plants resulting from the progeny of two consecutive back-crosses of each of the mutants with the wild-type were produced. These mutant lines, specifically lacking of plastidic GS, were further utilized to analyze different aspects of plastid GS functionality in *L. japonicus* plants as follows.

#### 2.2.1. Plastidic GS and Photorespiration Transcriptomics

In addition to its obligatory relationship with photosynthesis, the photorespiratory cycle interacts with several other primary and secondary pathways in the cell, including the Calvin cycle [71], nitrogen assimilation, respiration and one carbon metabolism [72] as well as redox signaling [73]. Given this intertwining of photorespiration with several other routes, it was interesting to study the effect of the impairment of the photorespiratory cycle on the overall cellular metabolism. For this reason, we made use of the *Ljgln2-2* mutant in order to determine if the effect of a transfer from suppressed to active photorespiratory conditions could affect the transcriptome of *L. japonicus*. The *Ljgln2-2* mutant was chosen since it lacked of any detectable levels both of plastidic GS activity and polypeptide. A preliminary study was carried out in leaves of WT and mutant plants that were grown for 45 days in CO_2_-enriched atmosphere and then transferred to normal air conditions. The transcriptomic analysis was carried under control conditions (CO_2_-enriched atmosphere) and after 2 days of exposition to normal air. Transcriptomic data were obtained using the recently developed Affymetrix GeneChip^®^ Lotus1a520343, that contains 52,749 unique probesets. A probeset is an oligonucleotide designed to measure the expression of a known or predicted sequence of mRNA. Several probesets may correspond to a same gene in such a way that most of *L. japonicus* gene transcripts are analyzed in a single DNA chip. Changes in gene expression between WT and *Ljgln2-2* plants were analyzed by a significance-based comparison applying a false discovery rate (FRD) < 0.05, using three different biological replicates.

The total number of probesets modulated by the 2-days shift to photorespiratory conditions was much higher for the mutant plants: 5,785 probesets were changed exclusively in the *Ljgln2-2* genotype compared to the 655 that changed exclusively in the WT (Table 1). In both genotypes, the number of down-regulated probesets was higher that the number of up-regulated ones. The higher number of probesets modulated for the mutant plants indicates that impairment of the photorespiratory cycle has a vast effect on leaf metabolism. On the other hand, 825 modulated probesets were common to both genotypes (Table 1). These probesets probably represents the core/common response of *Lotus japonicus* to photorespiration that is not dependent on the presence of plastidic GS.

A preliminary analysis of this dataset was carried out focusing on the ten more up-regulated and down-regulated genes (Table 2). The fold-change values for the top up- and down-regulated genes in the mutant were higher than the corresponding values for the WT plants. This probably reflects the higher levels of cellular stress present in this genotype under active photorespiration.

Two groups of genes were highly represented amongst the most induced in both genotypes: genes involved in flavonoid biosynthesis and in redox metabolism (Table 2). Three genes involved in flavonoid biosynthesis were highly induced in the WT (polyketide reductase, isoflavone reductase and 2-hydroxyisoflavanone synthase, corresponding to probesets chr2.CM0191.49.2, chr2.CM0249.88 and TM0802.13 respectively). Genes for flavonoid biosynthesis were also induced in the mutant, three of them encoding for chalcone synthase (probesets chr3.CM0590.56, chr2.CM0018.54 and Ljwgs_099009.1) in addition to the gene encoding for 2-hydroxyisoflavanone synthase that was induced also in the WT. Flavonoids are a vast class of secondary metabolites involved in an array of processes, including plant–pathogen interactions, pollination, light screening, seed development and allellopathy. Moreover, most flavonoids show an important anti-oxidant capacity [74]. Many of the genes involved in flavonoid biosynthesis are induced under biotic or abiotic stress. This is probably aimed to increase the production of flavonoids in order to scavenge the increased amount of reactive oxygen species generated under these conditions. The induction of flavonoids biosynthesis may then suggest that the transfer of both plant genotypes to active photorespiratory conditions may be associated to increased levels of oxidative stress. According to this idea, several genes related to redox metabolism were highly induced in both genotypes. A gene encoding for glutathione-S-transferase and one for alpha-dioxygenase were amongst the ten most induced both for WT and *Ljgln2-2*. Interestingly, the alpha-dioxygenase gene induced in both genotypes (probeset Ljwgs_903636.1) was similar to the alpha-DOX1 fatty acid dioxygenase from *Arabidopsis* (transcribed unit: At3g01420), that is involved in protection against oxidative stress and cell death [75].

Besides of the recycling of the glycolate-2-phosphate produced by the oxygenation reaction of the RUBISCO, the photorespiratory cycle also plays an important role in stress protection since it consumes ATP and reducing equivalent, preventing the over reduction of the photosynthetic electron transport which may result in the formation of reactive oxygen species [71]. It is then not surprising that the transfer of *L. japonicus* plants from photorespiratory-suppressed to photorespiratory-active conditions causes a response of flavonoid and redox metabolism. However, this response was not limited to the mutant with an impaired photorespiratory cycle but was also extended to the WT plants. This may indicate that, even in plants with normal levels of photorespiratory enzymes, the flux towards the photorespiratory pathway is associated with some levels of oxidative stress. Alternatively, it may also be possible that WT plants that have been grown under CO_2_-enriched atmosphere for more than one month may have lower-than-normal levels of some photorespiratory enzymes.

The two most up-regulated genes in the WT plants corresponded to transcription factors (TFs): a NAM, ATAF1/2 and CUC2 (NAC) domain TF (probeset chr2.TM0641.8) and an APETALA2 (AP2) and ethylene-responsive element binding proteins (EREBPs) family one (probeset chr5.CM0341.27). Two TFs were also induced in the mutant, a distinct domain one (probeset Ljwgs_036303.1) and one belonging to the myeloblastosis (Myb) family (probeset chr2.CM0250.2). The modulation of these transcription factors may be triggered either by the reactivation of photorespiration or by the diminishment of carbon availability in both genotypes. In the case of the mutant, changes could be expected also by the metabolic consequences of the impaired photorespiratory route. The fact that different TFs were modulated in WT and mutant plants suggests a role for plastidic GS in the regulation of photorespiratory metabolism. Many repressed genes encoded for hypothetical proteins of unknown function. Future experiments should be aimed to the characterization of these genes and their corresponding gene products.

In summary, the transcriptomic study carried out reveals that the transfer of *L. japonicus* plants from photorespiratory-suppressed to photorespiratory-active conditions results in important changes in gene expression. Some transcription factors, mainly of the Myb and NAC-domain families, were among the most affected transcripts. Other stress-related pathways associated to oxidative stress and flavonoid metabolism were also particularly modulated. This response was greatly enhanced as a consequence of deficiency in plastidic GS, thus indicating the crucial significance of plastid GS in photorespiratory metabolism transcriptomics.

#### 2.2.2. Plastidic GS and Nitrogen Nutrition

Plants can use various forms of combined nitrogen, most importantly the ions nitrate and ammonium (Figure 1). Despite of the fact that more energy is needed for the assimilation of nitrate, most plants prefer NO_3_^−^ over NH_4_^+^. With the exception of ammonium tolerant species, the availability of NH_4_^+^ alone as nitrogen source, as well as the internal production of NH_4_^+^ by processes like photorespiration [76] may result toxic to the plant. Notably, the toxic effect of external ammonium can be partially relieved by co-provision of nitrate, the so-called mixed nitrate plus ammonium nutrition. A fascinating and still poorly understood aspect of nitrogen nutrition is that in most cases the growth of a plant on NH_4_NO_3_ can surpass the maximal growth compared to either NO_3_^−^ or NH_4_^+^ alone. This relief of NH_4_^+^ toxicity by NO_3_^−^ may be related to a synergism between the signaling routes of NH_4_^+^ and NO_3_^−^ [77]. Moreover, several genes are modulated exclusively when the nitrogen source is NH_4_^+^, NO_3_^−^ or NH_4_NO_3_ [78].

As reported in this paper, the absence of plastidic GS in the *L. japonicus* mutant *Ljgln2-2* is associated with important transcriptomic changes under particular conditions like active photorespiration. We have examined if plastidic GS may also play a role in the transcriptional response of *L. japonicus* to the availability of different nitrogen sources. For this purpose, a study of the expression levels of key genes for nitrogen metabolism was carried out in leaves of WT and *Ljgln2-2* mutant plants that had been grown for 35 days in CO_2_-enriched atmosphere and different nitrogen sources (8 mM KNO_3_, 8 mM NH_4_Cl or standard Hornum mixed nutrition, which consisted of 5 mM KNO_3_ plus 3 mM NH_4_Cl). The expression of genes coding for glutamine synthetase, glutamate synthase, asparagine synthetase and glutamate dehydrogenase was monitored by qRT-PCR. Gene-specific oligonucleotides were synthesized based on sequences found in the available databases.

The comparison of the expression levels of the genes analyzed between WT and mutant plants is presented in Table 3 using a color code: boxes in green indicate higher expression in the WT than in the mutant while boxes in purple indicate higher expression in *Ljgln2-2*. Three different sequences corresponding to cytosolic GS were analyzed. Two of them (*LjGS1.1* and *LjGS1.3*) were more expressed in the mutant when the nitrogen source was either nitrate or ammonium. On the other hand, *LjGS1.3* was more expressed in the WT under mixed nutrition. *LjGS1.2* had a very different behavior with respect to the other two cytosolic GS genes as it was more expressed in the mutant but only under mixed nutrition. The two genes coding for plastidic GS and Fd-GOGAT (*LjGLN2* and *LjGLU1*) were more expressed in the mutant with nitrate or ammonium, but their transcription was higher in the WT under mixed nutrition. This result suggests that *LjGLN2* and *LjGLU1* are regulated in a common fashion, as confirmed by studies in other plants [79]. In the case of the two sequences found coding for NADH-GOGAT (*LjGLT1* and *LjGLT2*), the expression of both genes was higher in *Ljgln2-2* under mixed nutrition. Interestingly, the regulation of the two genes coding for asparagine synthetase in *L. japonicus* (*LjAS1* and *LjAS2*) was the same as *LjGLN2* and *LjGLU1*. Since plastidic GS and Fd-GOGAT are the enzymes that carry out the reassimilation of photorespiratory ammonium in the leaves, this parallelism may suggest a link between asparagine biosynthesis and the photorespiratory cycle. Evidence of an involvement of asparagine in photorespiration also comes from early studies using metabolite labeling [80]. Finally, four sequences corresponding to GDH were found in *L. japonicus* DNA sequences databases. The expression of *LjGDH3* was higher in the mutant plants under all the different nitrogen nutrition considered, while *LjGDH4* showed higher expression in the mutant only under mixed nutrition. On the other hand, the expression of *LjGDH1* and *LjGDH2* was undetectable in leaves (not shown). It is interesting to notice that for most genes the transcriptional effect of the deficiency in plastidic GS was very similar in the case of plants grown under NO_3_^−^ and NH_4_^+^ but different, and in several cases opposite to the effect produced in NH_4_NO_3_-grown plants. This was the case of the *LjGS1.3*, *LjGLN2*, *LjGLU1*, *LjAS1* and *LjAS2* genes. These results suggest a role for plastidic GS in the distinctive response to mixed nutrition of the nitrogen assimilatory genes in *L. japonicus*, and may imply a role of plastidic GS in the signaling events related to the presence of different nitrogen sources.

#### 2.2.3. Plastidic GS, Photorespiration and Nodulation

How photorespiratory metabolism affects nodulation has not been sufficiently studied. A recent study gives new insights into the influence of photorespiratory metabolism and GS2 on nodule function using photorespiratory mutants lacking GS2 isoenzyme [81]. It is generally assumed that GS1 is the GS isoenzyme responsible for primary assimilation of the NH_4_^+^ released by bacteroids [15]. Different approaches have been utilized to study the physiological impact of altered GS activity in the nodules, using transgenic plants showing a reduction or overexpression of GS1 enzyme activity [82,83]. The presence of plastid GS2 isoform in nodules has been reported for *Medicago truncatula* [84] and *L. japonicus* [81]. The studies carried out with GS2 mutants from *L. japonicus* determined that GS2 accounts for up to 40% of total nodule GS activity [81], a relatively unexpected result, since no decrease was observed in these mutant plants with regard to total GS activity from roots [24], a closely related organ to nodules. A comparative Western blot analysis of WT and GS2 mutant nodules showed a similar level of the GS1 polypeptide in WT, *Ljgln2-1* and *Ljgln2-2* genotypes, whereas the GS2 polypeptide was present in nodules of WT plants and was undetectable or only a faint band could be observed in both mutant genotypes. This indicated that the mutants were not only deficient in the GS2 isoform from leaves and roots as previously reported, but also in the GS2 isoform from nodules, which must be responsible for at least 40% of GS activity in this organ. So GS2 has a very significant contribution to the total levels of GS activity present in nodules of *L. japonicus* plants.

The fact that mutant plants lacking GS2 were still able to establish symbiosis and fix nitrogen in a similar way than WT indicated that the GS2 isoenzyme was not essential for nodulation and nitrogen-fixation processes under non-photorespiratory conditions. Therefore, it was confirmed that GS1 is sufficient for an efficient assimilation of ammonium derived from N_2_ fixation. Nevertheless, the influence of photorespiratory metabolism on nodulation could be also determined in WT and GS2 mutant plants, which were grown in CO_2_-enriched atmosphere (photorespiration-suppressed conditions) and then transferred to low CO_2_ atmosphere (photorespiration-active conditions) [81]. This transfer substantially affected the number of nodules obtained, the FW of these nodules, and the levels of ARA (acetylene reduction activity), which were highly reduced compared with the plants maintained under CO_2_ enrichment. At early developmental stages, mutant plants were affected in a very similar way to WT, thus indicating that the additional lack of GS2 did not have a significant influence on nodulation at this developmental stage. However, the transfer of the plants from high CO_2_ to air atmosphere at a later stage of plant development resulted in a dramatic reduction in nodule FW (40 to 60% decrease) and ARA activity (60 to 85% decrease) The mutant nodules were considerably more affected than the WT ones, indicating that GS2 deficiency affected nodule mass and function, particularly under photorespiratory active conditions. It was therefore demonstrated that the photorespiratory activity of the plant generates a negative influence in nodule formation, development, and function, at later stages of growth, particularly in GS2 deficient plants [81].

Sucrose is the predominant sugar detected in nodules of *L. japonicus* WT and GS2 mutant plants [81,85]. This carbohydrate is the first photosynthate supplied to nodules and, consequently, nitrogen fixation in legume nodules is highly dependent on the supply of sucrose delivered from the phloem [86]. When carbon levels are high, starch is the main stored compound. In fact, electron micrographs have previously suggested the presence of high starch content in nodules grown at elevated CO_2_ [87]. It has been reported that nodulated plants growing under atmospheric CO_2_ enrichment showed enhanced whole-plant growth and increased nodule biomass, and the nodules showed higher sugar and starch contents as well as enhancement of some activities related to nodule carbon metabolism and increased ARA [88–90]. These results indicated that under CO_2_ enrichment a high amount of carbon must be fixed and directed to nodules. In other studies, alfalfa plants with CO_2_ application tended to form fewer and bigger nodules, but in this case, the CO_2_ was applied around the root and nodule compartment [91] and not around the shoots, as in other studies. However, neither a short- nor a long term-effect on nodule ARA-specific activity has been observed in these plants [90,92]. By contrast, it has recently been described that ARA-specific activity increases in nodules of plants subjected to high CO_2_ treatment applied only to the root and nodule compartment [91]. In *L. japonicus* plants, carbohydrate analysis revealed a decrease of the levels of glucose, fructose, sucrose and starch in plant nodule extracts when plants growing in CO_2_-enriched atmosphere were transferred to low CO_2_ conditions [81]. These studies revealed that when photorespiration begins, the CO_2_ assimilation diminishes, as it is also the available photosynthate content that can be directed to nodules. The transfer to air conditions lead to alterations of carbon metabolism and therefore bacteroids were limited in carbon compound levels causing reduction of atmospheric nitrogen fixation, and thus indicating that the photorespiratory activity of the plant influences negatively the nitrogen fixation rate by limiting the carbon availability. The decrease of carbohydrate level, particularly starch and sucrose, in plant nodule extracts was more remarkable in nodules from mutant plants transferred to air (where a reduction of about 65 to 80% was observed) than in nodules from plants grown under CO_2_-enriched atmosphere. Interestingly, under photorespiration-suppressed conditions, the relative levels of sucrose were considerably lower (around 60% reduction) in nodules from mutant plants than in those from WT. These results revealed the existence of alterations in carbon metabolism of mutant nodules, under both photorespiratory- active and -suppressed conditions.

Recent reports established that antisense inhibition of NADH-GOGAT activity impairs carbon and nitrogen metabolism in alfalfa nodules [93]. The requirement of carbon skeletons for ammonium condensation and the supply of reducing equivalents as products of photosynthesis, respiration, and photorespiration pathways are well known [3,94]. It was also demonstrated that the general nitrogen nutritional status of *L. japonicus* plants can strongly affect the competence for nodule formation [95]. The reduction in number of nodules observed in the mutant plants after the shift to air conditions could be explained by the increased amount of non-assimilated ammonium representing an inhibitory signal for the nodule formation mechanism [95,96]. Studies with photorespiratory mutants containing normal levels of GS1 but specifically lacking of GS2 activity showed that the lack of GS2 substantially increased the negative effect of photorespiration on the nodulation process and nitrogen fixation in mutant plants. In addition, optical microscopy data revealed alterations in mutant nodules such as development restrictions, disappearance of starch granules in the non-infected cells, increase in size of vacuoles and appearance of regions with lower bacteroid density in the cytoplasm of infected cells, indicative of a cell lysis process [81].

In conclusion, the *Lotus-Rhizobium* symbiotic process was highly affected under active photorespiratory conditions. The studies with GS2 mutants deficient in photorespiratory ammonium assimilation emphasized how a “nitrogen” assimilation defect affected “carbon” metabolism and nodule function in *L. japonicus*. Interestingly, it was observed a clear-cut deficiency in carbon metabolism in mutant plants maintained under CO_2_-enriched atmosphere at later developmental stages, with a strong reduction in the sucrose nodule content as well as a significant decrease in starch, glucose and fructose levels compared with WT plants. Thus a role of GS2 in the C/N balance of *L. japonicus* plants, independent of the photorespiratory activity of the plant, can be established [81].

#### 2.2.4. Plastidic GS and Drought Stress Transcriptomics

Osmotic stress associated with salinity and drought is one of the most serious problems that reduces crop productivity world-wide. Legumes represent about one third of the world’s primary crop production and are a valuable protein source for both human and animal feeding. In particular, several species of the genus *Lotus* are used as pasture in temperate regions, where the plants can be exposed to sudden periods of drought. For this reason, the search for genes that may contribute to stress tolerance in *Lotus* is of particular significance. Such kinds of studies have been carried out in other plants using DNA chips, which permit the quantification of the transcription of thousand of genes. A great number of genes that are modulated by drought have been identified, particularly in the model plant *Arabidopsis thaliana*, but the function of only a limited number of them is known [97]. However, the recent availability of the Affymetrix Lotus1a520343 Genechip^®^ permitted to aboard the study of the transcriptomic response to drought also in this model legume. It was of particular interest to determine the drought stress transcriptome not only in WT plants but also in the *Ljgln2*-2 mutant. In fact, it has been described in other plants that GS may play an important role in the response to abiotic stress. For example, a role for cytosolic GS was clearly established in proline production [98]. On the other hand, overexpression of the plastidic GS isoform in non-legume plants like tobacco and rice resulted in enhanced tolerance to photooxidation [66] and to salt stress [99].

In order to study the role of *L. japonicus* plastidic GS in the response to drought stress, WT and *Ljgln2-2* mutant plants were submitted to 4 days of water deprivation. The *Ljgln2-2* mutant was chosen since it lacked of any detectable levels both of plastidic GS activity and polypeptide. WT and mutant plants were grown under a CO_2_-enriched atmosphere in order to avoid the concomitant effect of drought and photorespiration on the mutant. The lack of plastidic GS had several important effects in the response of the *Ljgln2-2* mutant to water deprivation. Mutant plants with the same level of hydric deficit as the WT showed higher thiobarbituric acid-reactive species (TBARS) content, indicating a higher level of oxidative membrane damage in *Ljgln2-2* under drought [100]. Moreover, the metabolism of proline, one of the most common compounds produced by plant cells in response to abiotic stress [101], was deeply altered in the mutant. In fact, *Ljgln2-2* plants accumulated less proline when compared to the WT ones at a similar hydric deficit levels and significant differences in the expression of the genes for proline metabolism were observed. These data established for the first time a link between plastidic GS and proline metabolism [100].

To gain further insight into the role of plastidic GS in the response to drought stress, the transcriptomes from leaves of WT and mutant plants after 4 days of water deprivation were compared with the transcriptomes of well-watered plants in order to identify the genes modulated by drought. Of the 52,749 probesets contained in the *Lotus japonicus* Genechip^®^, 538 corresponded to genes modulated by drought exclusively in the WT plants and 5,845 exclusively in the mutant, while 2,070 probesets were changed in both genotypes (Table 4).

A global analysis of the *Lotus* drought transcriptomic dataset was presented in a previous work [100]. Here, we will just focus on the top 10 genes that were most up-regulated and down-regulated in either WT or *Ljgln2-2* genotypes (Table 5). The sequences corresponding to the most highly modulated genes were used to search for similar ones in several databases in order to determine the function of each gene product. The genes identified in this way were related to different metabolic processes. For example, different genes involved in hormone metabolism were up-regulated: 1-aminocyclopropane-1-carboxylate (ACC) synthase (probeset chr1.TM1635.18), the enzyme that catalyzes the rate-limiting step in ethylene biosynthesis, was induced in both genotypes, suggesting increased ethylene biosynthesis under drought stress. The function of ethylene during drought is however a matter of controversy: ethylene alone seems to promote stomatal closure, a condition that reduces water loss, but it has also an inhibitory effect on the abscisic acid-stimulated stomatal closure suggestive of a yet little-understood crosstalk between different hormones during the stress response [102]. On the other hand, 12-oxyphytodienoate reductase, an enzyme involved in jasmonic acid biosynthesis, was induced only in the mutant (probeset TM0763.11).

Two different genes involved in the production of molecules with protective function were the most up-regulated in the two genotypes. In the WT, the most highly induced gene encoded for glutamate decarboxylase, an enzyme that produces γ-Aminobutyric acid (GABA). GABA is a compound that may have several roles in the response to stress such as regulation of the cellular pH, osmoregulation and/or protection against oxidative stress [103]. On the other hand, in the *Ljgln2-2* mutant the most up-regulated gene encoded for a thaumatin-like protein (probeset chr1.CM0012.67). Thaumatin-like proteins normally accumulate as a consequence of pathogens attack and exert an antibacterial/antifungal effect [104], but are also produced as a consequence of abiotic stress. Genes involved in proline metabolism were also induced in both genotypes [100], but were not present among the 10 top up-regulated ones.

Drought stress induced the modulation of genes related to cell wall metabolism. A gene encoding for pectinesterase, an enzyme involved in cell wall catabolism, was highly induced in both genotypes (probeset Ljwgs_036708.1). Moreover, a gene encoding for expansin, an enzyme involved in cell-wall loosening during the enlargement of plant cells, was highly down-regulated in the WT (probeset Ljwgs_056053.1). Taken together these results indicate that both WT and mutant genotypes are undergoing restructuration of the cell wall under conditions of water deprivation.

Several transcription factors (TFs) and genes related to signal transduction were among the most regulated by drought. Up-regulation of genes encoding for putative TFs was seen only in *Ljgln2-2*, where a NAC domain and a C_2_H_2_ family TFs were highly induced under drought conditions (probesets chr1.CM0104.32 and Ljwgs_020980.2). Since much more genes were modulated by drought in *Ljgln2-2* compared to the WT, these TFs may be involved in the unique transcriptional response of the mutant. An inositol-1,4,5-trisphosphate 5-phosphatase and a SYG1/Pho81/XPR1 (SPX) domain containing protein, both related to signal transduction, were induced in the WT and mutant respectively. Inositol-1,4,5-triphosphate is one of the major phospholipid-derived signaling molecule involved in signal transduction following osmotic stress [97].

Finally, other highly modulated genes encoded for redox-related enzymes like glutathione-S-transferase (probeset chr5.CM0909.59, induced in the WT) and glutaredoxin (probeset Ljwgs_040576.1). The gene coding for glutaredoxin was the first and third most repressed one in WT and *Ljgln2-2* respectively. Glutaredoxins are glutathione-dependent oxidoreductases that can regulate enzyme activity through reduction of enzyme disulfide bridges, and plays a crucial role in plant development and in the response to oxidative stress [105].

It is interesting to notice that the average fold-change of both up- and down-regulated genes was much higher in the case of the mutant (Table 5). This was also confirmed at the whole-transcriptome level [100]. This behavior indicates that the *Ljgln2-2* mutant may be suffering or perceiving a higher level of cellular stress than the WT after similar conditions of drought-stress. Besides of these differences, several of the top-modulated genes in WT and *Ljgln2-2* were related to similar functions like hormone metabolism, cell-wall metabolism and redox metabolism. This indicates that the modulation of these metabolic processes is part of the core response of *L. japonicus* to water deprivation and is independent from the presence of plastidic GS.

In conclusion, the analysis carried out here indicated that during water deprivation in *L. japonicus* a common response is triggered in both WT and *Ljgln2-2* including modulation of genes for hormone, cell-wall and redox metabolism as well as TFs and genes involved in signal transduction. However, much more genes were modulated in the mutant plants, and the extent of their modulation was more pronounced, reflecting the importance of plastidic GS in the response of the plant to drought stress. Some TFs were highly induced exclusively in the mutant, suggesting their possible involvement in the particular transcriptional response of the mutant to drought stress. The development of a TILLING reverse genetic tool [106] and, much more recently, of a population of insertion mutants created using the LORE1 endogenous retrotransposon [107] in *L. japonicus* may permit in the near future the evaluation of mutants of highly drought-responsive genes in this model legume.

#### 2.2.5. Co-Expression Analysis of *L. japonicus* Plastidic GS

The increasing amount of plant transcriptomic data available in public databases has boosted the number of studies that explore gene function using *in silico* approaches. An example of this consists in data mining for genes that have similar expression profiles (also called co-expression) under a large number of conditions. Such kind of analysis is of particular interest since it may reveal novel interconnections between different metabolisms in virtue of a similar expression of their genes. Based on the assumption that genes sharing the same expression profile are likely to be functionally related (the so called “guilt by association”), many genes have been associated in a wide range of organisms, including plants. The analysis of gene co-expression requires the availability of a large amount of high-quality data, generally from DNA microarrays under different experimental conditions. For this reason, co-expression studies have been carried out only in a limited number of model plants like *Arabidopsis*, barley, rice and poplar [108,109]. However, an online tool for co-expression analysis has been made available recently for *L. japonicus* (“The Lotus transcript profiling resource”, available online: http://cgi-www.cs.au.dk/cgi-compbio/Niels/index.cgi; accessed on 24 March 2012). This program utilizes the dataset from different arrays published by Høgslund *et al.* [110] that includes transcriptomic data from different tissues of WT and mutant *L. japonicus* plants.

**Figure 4:**
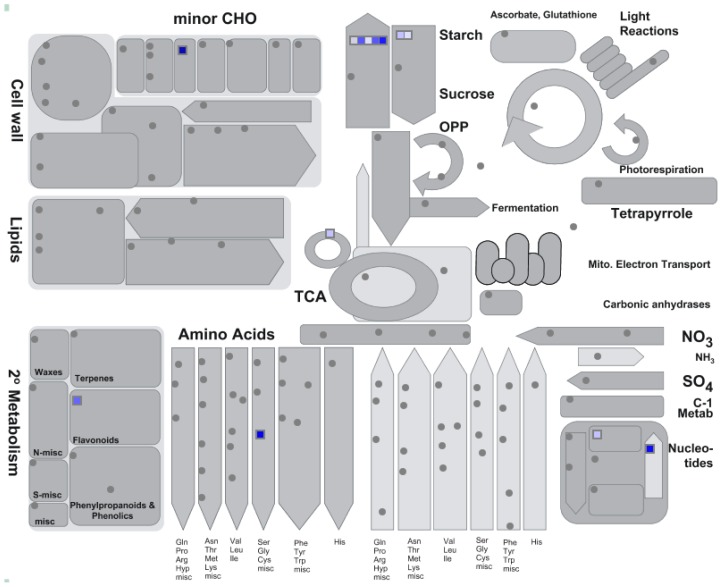
Co-expression analysis of plastidic GS. An overview of general metabolism was created using MapMan. Blue squares represent genes that are positively co-expressed (Pearson distance <0.3) with plastidic GS. The intensity of the blue color is proportional to the degree of correlation between a certain gene and the plastidic GS gene.

Using this resource, the top 100 genes whose transcription was positively correlated with the plastidic GS2 were identified. A Pearson distance cut-off value of <0.3 was applied in order to consider only highly positively co-expressed genes. This dataset was analyzed using the gene expression analysis feature of Genebins [111], a program that permits to detect if a certain functional category of genes (also called “BIN”) is statistically over-represented within a group. Using the default parameters given by the program (*p* < 0.05, Bonferroni correction), it was found that the functional category “starch synthase” was statistically (*p* = 3.8 × 10^−9^) more represented between this group of genes. This strong correlation of plastidic GS2 with starch metabolism was evident at a glance from the overview of general metabolism given by the MapMan program, where a total of seven probesets were assigned to the starch/sucrose pathway (five corresponding to starch synthase and two to beta amylase), with only six more probesets divided between the other metabolic routes (Figure 4). These results confirmed once again that plastidic GS2 is involved in the carbon metabolism of *L. japonicus* and, more specifically, suggested an involvement of this isoform in the starch and sucrose metabolism. This has been confirmed experimentally using the mutant *Ljgln2-2*, that showed substantially lower levels of starch and sucrose in the nodules than the WT affecting nodulation and nitrogen fixation [81] (see also Section 2.2.3). It has to be noticed that the transcriptomic dataset used to obtain the co-expression data presented here were mainly from non-photosynthetic tissues [110]. It may be then possible that the association of plastidic GS2 with starch and sucrose metabolism detected by the present study may be specific of roots and nodules. While plastidic GS2 is predominantly expressed in the green tissues of most plants, the presence of the GS2 polypeptide in non-photosynthetic tissues seems a typical feature of temperate legumes [25]. Nevertheless, a recent study correlated quite interestingly the rice plastidic GS2 gene expression with genes coding for the structural proteins of the photosystems and the enzymes of Calvin cycle and photorespiration [109].

Finally, we have to say that of the top 100 genes that were positively correlated with plastidic GS2 in co-expression analysis, 71 were defined either as unclassified with homologue or unclassified without homologue according to the KEGG-based ontology of Genebins, and 8 of them were involved in transcriptional regulation. Further work is still required to fully characterize the whole network of proteins that may be associated with plastidic GS2. As it has been reviewed in this paper, the plastidic GS2 from *L. japonicus* is clearly involved in many distinct processes. The everyday increasing amount of transcriptomic data, bioinformatics tools and mutant collections now available in *L. japonicus* will enable in the near future to better define the precise involvement of this important protein in the metabolic regulation of legume plants.

#### 2.2.6. Analysis of Heterozygous Plastid GS Mutant Plants

We have shown above that homozygous photorespiratory mutants affected in plastidic GS have been of fundamental importance to study plastidic GS functionality in *L. japonicus* plants as a result of of plastidic GS impairment. We have also analyzed the phenotypic behavior of *Ljgln2-1* and *Ljgln2-2* GS heterozygous mutant plants. While, in the long term, the homozygous photorespiratory mutants were not viable at ambient CO_2_ concentrations, heterozygous *Ljgln2-1* and *Ljgln2-2* mutants could be grown normally in air without showing any apparent stress symptom or ammonium accumulation, thus confirming the recessive character of the mutant traits. Quantification of the GS protein and enzyme activity levels in the heterozygotes led to quite interesting findings. It was observed that heterozygote *Ljgln2-2* had intermediate levels of total GS activity and plastidic GS protein, as it was expected according to the behavior of similar plastidic GS mutants from barley [23]. In contrast, the heterozygote *Ljgln2-1* showed normal levels of plastidic GS polypeptide and cytosolic GS, but completely lacked of plastidic GS activity, similarly to the homozygous mutant plants. This interesting behavior indicated that *Ljgln2-1* mutation is recessive for the air-sensitivity, ammonium accumulation and plastidic GS polypeptide level phenotypes, but it is a negative dominant mutation in regard to plastidic GS activity. This means that the presence of inactive mutant subunits of plastidic GS is able to avoid the formation of active GS oligomers even in the presence of wild-type active subunits of plastidic GS. The concept of negative dominant mutation was first defined by Herskowitz [112] on its classic paper in *Nature*. Curiously, in this paper it was predicted that the GS enzyme, in virtue of its multimeric nature, might be particularly sensitive to the presence of negative dominant mutations in the GS genes. However, how can be explained the air-insensitivity phenotype showed by heterozygous *Ljgln2-1*, which has normal levels of the GS2 polypeptide but apparently no GS2 activity? One possible explanation may be that heterozygous *Ljgln2-1* has almost undetectable levels of GS2 activity, but some tiny residual activity that could be sufficient to efficiently reassimilate the ammonium produced by photorespiration. This hypothesis implies that plastidic GS is present in a large excess in the chloroplasts of *L. japonicus*. Considering that photorespiration is a high-flux pathway [73], and that the ammonium assimilated through photorespiration may be 10 times higher that the one trough primary assimilation [4], it seems improbable that a residual amount of activity may be sufficient to reassimilate all the ammonium produced by glycine decarboxylation when the heterozygous *Ljgln2-1* plants are grown under normal air conditions. An alternative and more likely explanation may be that in the heterozygote *Ljgln2-1* plants different types of WT-mutant hetero-oligomers may be predominantly formed that could be at least partially active *in vivo*, but their GS enzyme activity may be lost when extracted from the cell in order to obtain crude extracts for the determination of the *in vitro* GS activity. It is possible that some post-translational modification of Lotus plastidic GS is essentially required for enzyme activity *in vivo* and may be altered as a result of *Ljgln2-1* mutation when preparing the crude extracts from heterozygous plants.

## 3. Conclusions

In this paper we have described the recent progress made in our laboratory on the structure-function and functional genomics of glutamine synthetase, a crucial enzyme of nitrogen metabolism in legumes. Main advances produced can be summarized as follows: (1) We have shown that the quaternary structure of plant GS depends on metal cofactors and, in some cases, is highly unstable and can be disassembled by single-point mutations resulting in a complete loss of enzymatic activity; (2) We report the existence of different activity forms of plant GS with different affinity towards glutamate and other types of evidence which are all suggestive of post-translational modifications of this protein; (3) We also show the important significance of plastidic GS2 isoform in various processes such as photorespiration, nitrogen nutrition, nodulation, and drought-stress. In fact, it is described how the lack of GS2 produces major changes in the transcriptome of *L. japonicus* plants and, in many cases, changes in carbon metabolism.

## Figures and Tables

**Table 1:** Probesets modulated by 2-days transfer from CO_2_-enriched (suppressed photorespiration) to normal air (active photorespiration) atmosphere in the leaf transcriptomics of either WT or *Ljgln2-2* mutant plants.

	WT only	Shared	*Ljgln2-*2 Only
**Total Probesets**	655	825	5785
**Up-Regulated**	155	384	2470
**Down-Regulated**	500	441	3315

**Table 2:** Top 10 genes up- or down-regulated by active photorespiration in WT and *Ljgln2-2*.

WT			

Probeset	log_2_ FC	Description	Similar to
**Up-Regulated**			
chr2.TM0641.8	1.98	NAC domain protein	AT1G69490
chr5.CM0341.27	1.96	AP2-EREBP Transcription factor	AT3G23240
chr2.CM0191.49.2	1.91	Polyketide reductase	AT1G59960
Ljwgs_093636.1	1.87	Alpha-dioxygenase	AT3G01420
Ljwgs_050995.1	1.77	Methyltransferase protein	AT3G11480
Ljwgs_080010.1	1.73	Pleiotropic drug resistance protein	AT1G15520
chr4.CM0256.39	1.73	Cytochrome P450	AT4G37370
chr2.CM0249.88	1.72	Isoflavone reductase	AT4G39230
TM0802.13	1.69	2-Hydroxyisoflavanone synthase	AT5G06900
Ljwgs_018999.1	1.64	Glutathione S-transferase	AT2G29420
**Down-regulated**			
Ljwgs_108871.1	−2.74	Hypothetical protein	AT3G02550
Ljwgs_035693.2	−2.46	Hypothetical protein	AT3G20810
Ljwgs_089359.1.1	−2.02	Early flowering 4 protein	AT2G40080
chr3.CM0711.3.2	−1.93	Unknown protein	AT4G10270
chr2.CM0191.60	−1.83	Nlj21	-
Ljwgs_080939.1	−1.81	Beta-glucosidase like protein	AT2G44480
chr3.TM0426.3	−1.75	Hypothetical protein	AT5G22580
chr1.CM0398.23.1	−1.66	Gibberellin induced protein	AT1G74670
chr3.CM0155.27	−1.64	Peroxidase	AT1G05260
TC17223	−1.64	Hypothetical protein	-

**WT**			

***Ljgln2-2***			
**Probeset**	**log_2_** **FC**	**Description**	**Similar to**

**Up-Regulated**			
Ljwgs_044797.1	5.37	60S ribosomal protein	AT1G26910
chr3.CM0590.56	4.52	Chalcone synthase	AT5G13930
Ljwgs_036303.1	4.43	NAC domain protein	AT4G27410
TM0802.13	4.35	2-Hydroxyisoflavanone synthase	AT5G06900
chr2.CM0250.2	4.34	MYB transcription factor	AT4G37260
gi45637799	4.26	Hypothetical protein	-
chr2.CM0018.54	4.19	Chalcone synthase	AT5G13930
Ljwgs_099009.1	4.06	Chalcone synthase	AT5G13930
Ljwgs_093636.1	4.04	Alpha-dioxygenase	AT3G01420
chr5.CM0909.51	4.03	Glutathione S-transferase	AT2G29420
**Down-regulated**			
chr1.CM0001.63	−4.66	Probable 2-Isopropylmalate synthase	AT1G74040
Ljwgs_091497.1	−2.97	Myo-inositol-1-phosphate synthase	AT5G10170
TM0810.14	−2.88	Cytochrome P450	AT2G45550
chr1.CM0398.23.1	−2.80	Gibberellin regulated protein	AT1G74670
TM1614.14.1	−2.60	Hypothetical protein	AT1G59960
Ljwgs_028558.1	−2.55	Pectate lyase	AT4G24780
Ljwgs_062989.1	−2.54	Terpene synthase	AT4G16730
chr3.CM0142.55	−2.52	Hypothetical protein	AT5G20190
chr1.CM0001.70.2	−2.45	Hypothetical protein	AT5G13750
Ljwgs_043433.1	−2.35	Benzoyl transferase	AT5G17540

The table shows the probesets corresponding to the 10 genes that showed higher extent of modulation (FDR < 0.05) in their transcript levels in the leaves of *L. japonicus* plants after the transfer for 2 days from 0.7% (v/v) CO_2_ (suppressed photorespiration) to normal air (active photorespiration) conditions as determined by transcriptome analysis using Affymetrix genechips. The log_2_ of the fold-change is indicated together with the description of the most probable match and the most similar Arabidopsis gene. The following databases were used for the search: the Kazusa DNA research institute (Available online: http://www.kazusa.or.jp/lotus/blast.html; accessed on 16 March 2012), Non-redundant protein sequences (Available online: http://blast.ncbi.nlm.nih.gov/; accessed on 16 March 2012), TAIR (Available online: www.arabidopsis.org; accessed on 16 March 2012) and the Legume transcription factor database (Available online: http://legumetfdb.psc.riken.jp/index.pl; accessed on 16 March 2012).

**Table 3:** Changes in the transcript levels for different isoforms of GS, GOGAT, AS and GDH in leaves of WT and *Ljgln2-2* mutant plants grown in the presence of different nitrogen sources. Transcript levels were determined by qRT-PCR using three independent biological replicates. Data are reported as the ratio of transcript levels between *Ljgln2-2* and WT plants, previously standardized to housekeeping genes. Genes that are more expressed in the mutant than in the WT, according to Student’s t test (*p* < 0.05), are highlighted in purple, while boxes in green indicate significantly higher expression in the WT.

Name	Probeset	Nitrogen Source		

		NO_3_^−^	NH_4_^+^	NH_4_NO_3_
*LjGS1.1*	TM0053.11	2.71	2.69	0.93
*LjGS1.2*	gi1246767	0.95	0.84	1.88
*LjGS1.3*	Ljwgs_019428.1	6.22	3.94	0.22
*LjGLN2*	gi18266052	8.99	4.96	0.19
*LjGLU1*	chr1.CM0009.24	4.77	2.64	0.25
*LjGLT1*	Ljwgs_035611.1	0.71	1.40	1.36
*LjGLT2*	Ljwgs_037992.1	1.06	1.70	1.23
*LjAS1*	gi897770	4.91	2.58	0.30
*LjAS2*	gi897772	4.57	3.64	0.73
*LjGDH3*	Ljwgs_035272.1	2.32	1.73	1.46
*LjGDH4*	Ljwgs_009442.1	1.09	1.04	1.13

**Table 4:** Probesets elicited by 4 days of water deprivation in the leaf transcriptomics for either WT or *Ljgln2-2* mutant plants.

	WT Only	Shared	*Ljgln2-*2 Only
**Total Probesets**	538	2070	5845
**Up-Regulated**	207	946	3636
**Down-Regulated**	331	1124	2209

**Table 5:** Top 10 genes up- or down-regulated by drought in WT and *Ljgln2-2*.

WT			

Probeset	log_2_ FC	Description	Similar to
**Up-Regulated**			
TC11101	4.83	Glutamate decarboxylase	AT5G17330
chr1.TM1635.18	4.77	ACC synthase	AT3G61510
Ljwgs_047159.1	4.61	STIG1-related protein	AT1G11925
chr5.CM0909.59	4.47	Glutathione S-transferase	AT2G29420
chr1.CM0141.2	4.37	Nitrate/peptide transporter	AT1G32450
Ljwgs_036708.1	4.32	Pectinesterase	AT2G45220
chr4.CM0429.5	4.32	Mitochondrial inner membr. translocase	AT4G16160
chr5.CM0089.120	4.15	Inositol-1,4,5-trisphosphate 5-phosphatase	AT1G47510
chr5.CM0148.50.2	4.11	Cytochrome P450	AT5G52400
TM0763.11	4.08	12-oxophytodienoate reductase	AT2G06050
**Down-regulated**			
Ljwgs_040576.1	−4.15	Glutaredoxin	AT5G18600
Ljwgs_056053.1	−4.15	Alpha-expansin family protein	AT2G39700
Ljwgs_006332.1	−3.92	Hypothetical protein	AT1G30260
chr1.BM1732.18	−3.82	Hypothetical protein	AT3G11210
BM0976.11	−3.78	Hypothetical protein	AT2G01050

**WT**			

Ljwgs_028040.1	−3.74	Ammonium transporter	AT4G13510
chr1.CM0233.42	−3.47	Nucleic acid binding protein	AT1G52950
chr6.TM1374.27	−3.41	hydroxycinnamoyl-CoA shikimate/quinate hydroxycinnamoyltransferase-like	AT2G19070
Ljwgs_016759.2	−3.41	Chloride channel protein	AT5G40890
TM1490.11	−3.35	MYB transcription factor	AT2G21650
***Ljgln2-2***			

**Probeset**	**log_2_** **FC**	**Description**	**Similar to**

**Up-Regulated**			
chr1.CM0012.67	8.20	Thaumatin-like protein	AT1G20030
Ljwgs_075692.1.1	7.87	GDSL esterase/lipase	AT1G29660
chr3.TM1465.12	7.50	SPX domain-containing protein	AT2G45130
chr1.TM1635.18	7.34	ACC synthase	AT3G61510
Ljwgs_036708.1	6.93	Pectinesterase	AT2G45220
chr2.CM1150.57	6.84	Metalloendoproteinase	AT1G70170
chr1.CM0104.32	6.84	NAC domain transcription factor	AT3G04070
Ljwgs_020980.2	6.80	C2H2 transcription factor	AT2G37430
Ljwgs_047159.1	6.73	STIG1-related protein	AT1G11925
Ljwgs_145133.1	6.72	Late embryogenesis abundant (LEA)	AT3G53040
**Down-regulated**			
chr1.BM1732.18	−8.81	Lipase/hydrolase protein	AT3G11210
chr2.CM0249.113	−7.27	Myb familily transcription factor	AT2G19510
Ljwgs_040576.1	−6.77	Glutaredoxin	AT5G18600
Ljwgs_073999.0.1	−6.70	Cytochrome P450	AT1G24180
Ljwgs_052903.1	−6.12	Lipoxygenase	AT1G55020
Ljwgs_058749.1	−5.73	Beta-glucosidase	AT5G42260
Ljwgs_127990.1	−5.62	Myb-related transcription factor	AT4G39250
TM0990.31.1	−5.59	Hypothetical protein	-
chr3.CM0590.43	−5.55	Hypothetical protein	AT4G27450
Ljwgs_091781.1	−5.45	Lipoxygenase	AT1G55020

The table shows the probesets corresponding to the 10 genes that showed higher extent of modulation (FDR < 0.05) in their transcript levels in the leaves of *L. japonicus* plants after 4 days of water deprivation as determined by transcriptome analysis using Affymetrix genechips. The log_2_ of the fold-change is indicated together with the description of the most probable match and the most similar Arabidopsis gene. The following databases were used for the search: the Kazusa DNA research institute (Available online: http://www.kazusa.or.jp/lotus/blast.html; accessed on 16 March 2012), Non-redundant protein sequences (Available online: http://blast.ncbi.nlm.nih.gov/; accessed on 16 March 2012), TAIR (Available online: www.arabidopsis.org; accessed on 16 March 2012) and the Legume transcription factor database (Available online: http://legumetfdb.psc.riken.jp/index.pl; accessed on 16 March 2012).
